# Combined Effects of Inoculation Level and Sequence on Biochemical and Microbiological Characteristics of Probiotic Doogh 

**Published:** 2013

**Authors:** Mohammad Rahmati Roudsari, Sara Sohrabvandi, Aziz Homayouni Rad, Amir Mohammad Mortazavian

**Affiliations:** a *Skin Research Center, Vice Presidency of Research and Technology, Shahid Beheshti University of Medical Sciences, P.O. Box 19395-4741, Tehran, Iran.*; b *Department of Food TechnologyResearch, National Nutrition and Food Technology Research Institute, Shahid Beheshti University of Medical Sciences, P.O. Box 19395-4741, Tehran, Iran.*; c *Department of Food Science and Technology, Faculty of Nutrition, Tabriz University of Medical Sciences, Tabriz, Iran.*; d *Department of Food Science and Technology, National Nutrition and Food Technology Research Institute, Faculty of Nutrition Sciences, Food Science and Technology, Shahid Beheshti University of Medical Sciences, P.O. Box 19395-4741, Tehran, Iran. *

**Keywords:** Doogh, Inoculation, Probiotic, Viability

## Abstract

The combined effects of inoculation level (4 or 8-fold compared to standard inoculation) and sequence (standard inoculation before fermentation and 3-fold inoculation at the end of fermentation=1+3, Two-fold inoculation before fermentation and the same at the end of fermentation=2+2, 2+6, 4-fold before fermentation=4, 4+4, and 8) of culture inoculum containing probiotics on biochemical and microbiological characteristics of probiotic Doogh during fermentation and over 21 days of refrigerated storage (4˚C) were investigated. The probiotic microorganisms were *L. acidophilus *LA-5 and *Bifidobacterium lactis *BB-12. Overall, the treatments 8, 4 and 4+4 resulted in the highest viability at the end of fermentation as well as at early days of refrigerated storage. During the second half of cold storage period, the greatest viability of probiotics was related to the treatment 2+6. The treatment ‘8’ showed the shortest incubation time as well as the highest pH drop rate and acidity increase rate during fermentation and over the storage period.

## Introduction

In food industry ‘Probiotics’ are known as live and active microorganisms incorporated in food products with an intention of remaining viable until the end of storage time. In fermented milk, probiotics might be added before fermentation to take part in fermentation along with the yogurt bacteria or might be added at the end of fermentation as a ‘carry-through’ (1). Therefore, from industry point of view, each probiotic strain supplemented into food product must be viable to a minimum standard level in order to exert its positive effects on the health of the host. The minimum level of viability for probiotics not presently follows a global national standard and each country offers its own amount, although some international standards such as ‘Codex Alimentations Commission’ (CAC) present their levels as recommendations in guidelines (2). As a general accepted level, the probiotics must be alive in the product at minimum viable numbers of 107 cfu/mL (3, 4). The minimum viability considers survival drop of probiotics during the production and storage of product as well as during the delivery through the gastrointestinal tract up to the intestine (2). 

Various factors affect the viability of probiotic bacteria during processing or storage of fermented milks such as pH, titrable acidity, molecular oxygen, redox potential, hydrogen peroxide, bacteriocins, biorelationships among starter bacteria and microbial competitions, short chain fatty acids, some flavoring agents, anti-microbial preservatives, packaging materials and conditions, level and proportion of inoculation, step-wise/stagewise fermentation, microencapsulation, supplementation of milk with nutrients, incubation temperature, storage temperature, carbonation, addition of salt, sugar and sweeteners, cooling level of product (2, 5-9) and even music waves (10). Between, incubation parameters (incubation level and incubation sequence compared to the standard levels offered by manufacturers’ instruction) have significant and considerable impacts in probiotic viability and a few works in fermented milks have been performed to consider this effect (11, 12). However, in none of the studies, the combined effects of inoculation level and sequence (before or after fermentation) on biochemical and microbiological characteristics of fermented milks have been considered. In this research, mentioned factors are investigated for Doogh, the Iran national dairy drink and the popular and high-consumed product in Iran.

## Experimental

*Starter culture*

The DVS (Direct-in-Vat-Set) pouches of commercial lyophilized mixed culture that commercially known as ABY-type including yogurt bacteria (mixed culture of *Streptococcus thermophilus *and *Lactobacillus delbrueckii *ssp. *bulgaricus*), *L. acidophilus *LA-5 and *Bifidobacterium lactis *BB-12 were supplied by Chr-Hansen (Horsholm, Denmark). These starter cultures are widely used by the dairy industry to produce probiotic fermented milk products. The cultures were maintained according to manufacturer’s instructions at -18˚C, until used.

*Sample preparation and study design*

Doogh milk with 4% of milk solid non-fat was made by reconstitution of skim milk powder (Pak Co., Tehran, Iran). Then, the mixture was incorporated with 0.7% (m/m) industrial sodium chloride. The milk was subjected to heat treatment (90°C/15 min) and after cooling down to fermentation temperature (40°C), samples with primary fermentation were inoculated with ABY-type culture in different states: according to the manufacturer’s instruction as standard inoculation (I), 2-fold of ‘I’ (2I), 4-fold of ‘I’ (4I), or 8-fold of ‘I’ (8I). Fermentation was carried out until a pH of 4.2±0.02 was reached. During fermentation, the pH drop, acidity increase and redox potential increase was recorded every 30 min. At the end of fermentation, fermentation time as well as the viability of probiotics were recorded and assessed. For treatments with sequential inoculation, the secondary inoculations were carried out: 3-fold following previous 1-fold=(1+3)I, 2-fold following the previous 2-fold= (2+2)I, 6-fold following the previous 2-fold= (2+6)I, and 4-fold following the previous 4-fold= (4+4)I. Biochemical parameters as well as the viability of probiotics were measured during 21 days of cold storage (4˚C). The concentration of acetic acid was also determined in all treatments at the end of fermentation and at the end of storage time. In each Doogh sample, 0.02% (m/m) of mint essence (Döhler, Germany) was added before refrigerated storage and finally, the sealed bottles of Doogh were stored under refrigeration temperature. 

*Microbiological analysis*

MRS-bile agar medium (MRS agar by Merck, Darmstadt, Germany and bile by Sigma-Aldrich, Inc., Reyde, USA) was used for the selective enumeration of *L. acidophilus *and bifidobacteria in ABY culture composition according to Sohrabvandi *et al. *(13). The plates were incubated aerobically and anaerobically at 37°C for 72 h. Anaerobic conditions were produced using the GasPac system (Merck, Darmstadt, Germany). 

*Chemical analysis*

pH and redox potential values of the samples were measured at room temperature using a pH meter (MA235, Mettler, Toledo, Switzerland). The titrable acidity was determined after mixing 10 mL of sample with 10 mL of distilled water and titrating with 0.1 N NaOH using 0.5% phenolphthalein. Various biochemical parameters were defined and determined as follows (5, 8): 

Mean pH drop level (mpH-DR) = (final pH value – initial pH value) / incubation time [pH value/min] 

Mean acidity increase rate (mA-IR) = (final acidity value – initial acidity value) / incubation time [Dornic degree/min] Mean redox potential increase level (mRP-IR)= (final value – initial value) / incubation time [mV/min] 

Mean redox potential increase level (mRP-IR) = (final value – initial value) / incubation time [mV/min] 

Quantification of lactic and acetic acids was carried out by High Performance Liquid Chromatography (CE 4200- Instrument, Cecil, Milton Technical Center, Cambridge CB46AZ, UK) according to Mortazavian *et al. *(14). Briefly, for extraction of acids, 4.0 g of sample was diluted to 25 mL with 0.1 N H_2_SO_4_, homogenized and centrifuged at 5000 g for 10 min. The supernatant was filtered through Whatman #1 filter paper and through a 0.20 μm membrane filter, and was immediately analyzed. A Jasco UV-980 detector and a Nucleosil 100-5C18 column (Macherey Nagel, Duren, Germany) were used. The mobile phase was 0.009 N H_2_SO_4 _at a flow level of 0.5 mL min-1. The wavelength of detection was optimized at 210 nm. The standard solutions of lactic and acetic acids (Merck, Darmstadt, Germany) were prepared in distilled water. The retention times for lactic and acetic acids were 3.45 and 3.58 min and the standard curve regression coefficients were 0.989 and 0.991, respectively. 

*Statistical analysis*

All results were an average of three replicate determinations and the significant differences among the means were analyzed using the one-way and two-way ANOVA test (based on the complete randomized design-full Factoriel test design) from Minitab software (Version 13, 2002).

## Results and Discussion

*Changes in pH, titrable acidity and redox potential*

**Figure 1:**
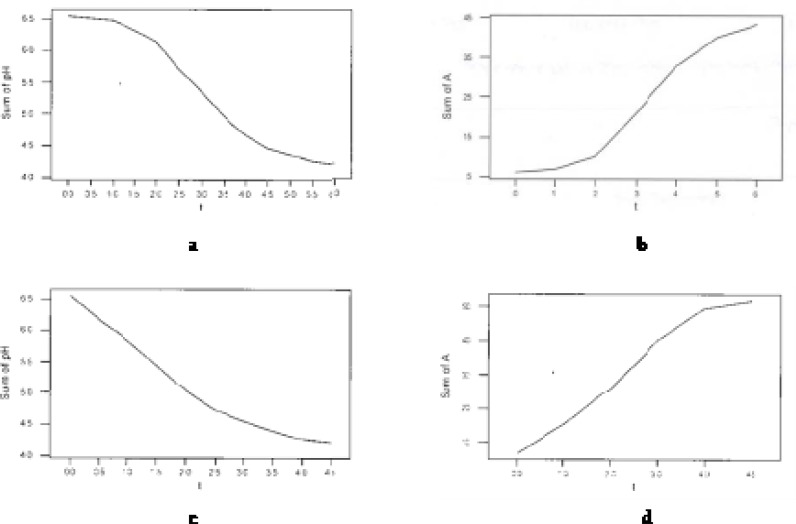
pH drop and acidity increase during fermentation in I (a,b) and 8I (c,d) treatment

Figure 1 shows pH drop and acidity increase during fermentation in I and 8I treatments. As expected, inoculation level (I, 2I, 4I or 8I) considerably affected the trends of acidity increase and pH decline during fermentation (Figure 1). 

As indicated, no lag phase at the start of pH drop and acidity increase curves were observed in 8I treatment compared to ‘I’. The reason is considerable higher growth and activity of starter cultures in treatments with greater inoculation level. Mentioned property is also represented in Table 1 that shows pH drop, acidity increase and redox potential increase levels of treatments per time intervals during fermentation. As appeared, in pH decrease trend for 8I, only two break points in fermentation time (h 2.5 and h 4) was observed compared to three (h 0.5, h 2 and h 4.5) in I. The lag and pre-log phases in I ended at h 2 of fermentation (according to the both trends of pH decline and acidity increase in Table 1) with the lowest mean pH drop level and mean acidity increase level, whilst these stages were not seen for 8I and the starter bacteria are in log phase from very initial minutes of fermentation. 

**Table 1:** pH drop, acidity increase and redox potential increase levels of treatments per time intervals during fermentation period until final pH of 4.2*

**Treatments**	**pH drop per time intervals**				**Titrable acidity increase per time intervals**					**Redox potential increase per time intervals**					
** I/(1+3)I	0.0-0.5	0.5-2.0	2.0-4.5	4.5-6.0	0.0-1.0	1.0-2.0	2.0-4.0	4.0-5.0	5.0-6.0	0.0-1.0	1.0-2.0	2.0-4.0	4.0-5.5	5.5-6.0	
	0.001^d^	0.004^b^	0.011^a^	0.003^c^	0.02^d^	0.05^c^	0.19^a^	0.12^b^	0.05^c^	0.06^d^	0.36^b^	0.75^a^	0.28^c^		0.05^d^
I(2+2) (2+6)I	0.0-0.5	0.5-2.0	2.0-4.5	4.5-6.0	0.0-1.0	1.0-2.0	2.0-4.0	4.0-5.0	5.0-6.0	0.0-0.5	0.5-2.0		2.0-4.5		4.5-6.0
	0.002^cd^	0.007^b^	0.011^a^	0.003^c^	0.05^c^	0.10^b^	0.16^a^	0.11^b^	0.04^c^	0.13^c^	0.41^b^		0.65^a^		0.08^d^
4I/(4+4)I	0.0-0.5	0.5-2.5	2.5-4.0	4.0-5.5	0.0-2.0		2.0-4.0		4.0-5.5	0.0-0.5	0.5-2.5		2.5-4.5		4.5-5.5
	0.003^c^	0.011^a^	0.008^b^	0.002c^d^	0.08^b^		0.19^a^		0.03^c^	0.27^c^	0.65^a^		0.40^b^		0.12^d^
	0.0-2.5		2.5-4.0	4.0-4.5	0.0-1.0		1.0-4.0		4.0-4.5	0.0-2.5		2.5-4.0			4.0-4.5
	0.012		0. 0 0 5^b^	0.001^c^	0.11^b^		0.20^a^		0.06^c^	0.11^c^		0.32^b^			0.74^a^

*Means in same rows shown with different letters are significantly different (p < 0.05).

** I = standard inoculation, 4I = 4-fold inoculation, 8I = 8-fold inoculation, (1+3)I = standard inoculation before fermentation (primary inoculation) and 3-fild inoculation at the end of fermentation (secondary inoculation), and *etc*.

Table 2 represents acidity increase level of treatments per pH drop intervals during fermentation. As can be seen, in treatment I, the log phase (highest mean acidity increase=0.19 ˚D/min) was placed through the pH range of 6.14-4.66, whilst this range for the treatment 8I was 5.83-4.26. In parallel to increase in inoculation level, the synergistic relationship among starter bacteria is enhanced leading higher pH drop rate and acidity increase rate as well as the shorter incubation time. 

**Table 2:** Acidity increase level of treatments per pH drop intervals during fermentation period until final pH of 4.2*

Tretments	Acidity increase levels per pH drop intervals				
** I/(1+3)I	6.54-6.47	6.47-6.14	6.14-4.66	4.66-4.35	4.35-4.22
	0.02^a^	0.05^c^	0.19^a^	0.12^b^	0.05^c^
(2+2)I/(2+6)I	6.62-6.41	6.41-5.95		5.95-4.31	4.31-4.21
	0.04^cd^	0.11^c^		0.14^a^	0.05^c^
4I/(4+4)I	6.52-5.59			5.59-4.44	4.44-4.21
	0.08^b^			0.19^a^	0.03^c^
8I	6.54-5.83			5.83-4.26	4.26-4.21
	0.11^b^			0.20^a^	0.06^c^

Table 3 shows mean pH drop rate, mean acidity increase rate and mean redox potential increase rate in treatments during fermentation. The same parameters during 21 days of refrigerated storage are shown in Table 4. 

**Table 3:** Mean pH drop rate, mean acidity increase rate, mean redox potential increase rate and incubation time in treatments at the end of fermentation*

**Parameters**				
**Treatment**	mpH-DR** **(pH/min)**	**mA-IR** **(°D/min)**	**mRP-IR** **(mV/min)**	**Fermentation time (min)**
I/(1+3)I ***	0.006^c^	0.10^bc^	0.40^bc^	360^a^
(2+2)I/(2+6)I	0.006^c^	0.10^bc^	0.42^b^	360^a^
4I/(4+4)I	0.007^b^	0.11^b^	0.43^b^	330^b^
8I	0.009^a^	0.16^a^	0.53^a^	270^c^

*Means in same columns shown with different letters are significantly different (p < 0.05).

**mpH-DR = pH drop level, mA-IR = acidity increase level, mRP-IR = redox potential increase level.

*** I = standard inoculation, 4I = 4-fold inoculation, 8I = 8-fold inoculation, (13+)I = standard inoculation before fermentation (primary inoculation) and 3-fild inoculation at the end of fermentation (secondary inoculation), and *etc.*

**Table 4:** Mean pH drop rate, mean acidity increase rate, mean redox potential increase rate, and incubation time and acetic acid percent in treatments at the end of fermentation*

**Parameters**									
**Treatment**	mpH-DR** **(pH/day)**	**mA-IR** **(°D/day)**	**mRP-IR** **(mV/day)**	**Final pH**	**Final acidity ** **(°D)**	**Final RP ** **(mV)**	**Acetic acid** **(%)**		
							**d 0**	**d 21**	
I***	0.002^f^	0.23^d^	0.20^d^	4.17^a^	47.9^d^	184.5^b^	0.03^d^		0.04^d^
(1+3)I	0.003^e^	0.44^c^	0.30^c^	4.14^b^	52.3^bc^	186.5^ab^	0.03^d^		0.04^d^
(2+2)I	0.004^d^	0.46^c^	0.40^b^	4.13^b^	52.6^bc^	189.1^a^	0.05^c^		0.06^c^
(2+6)I	0.005^c^	0.48^bc^	0.42^b^	4.10^c^	53.4^b^	189.1^a^	0.05^c^		0.06^c^
4I	0.004^d^	0.45^c^	0.41^b^	4.12^bc^	52.7^bc^	189.0^a^	0.08^b^		0.09^b^
(4+4)I	0.006^b^	0.50^b^	0.43^b^	4.09^c^	53.9^b^	189.4^a^	0.08^b^		0.10^b^
8I	0.007^a^	0.80^a^	0.48^a^	4.07^cd^	60.0^a^	190.4^a^	0.11^a^		0.12^a^

According to Table 3, the greatest mean pH drop rate and acidity increase rate were related to the 8I, with remarkable difference compared to others. No significant different was observed between the treatments with standard inoculation (I) and two-fold inoculation (2+2 or 2+6). Therefore, increasing inoculation rate by two times did not enough to make significant differences in bacterial growth and activity during fermentation. 8I had the shortest incubation time, whilst no significant different was seen between ‘I’ and (2+2)I or (2+6)I. 

Corresponding Table 4, during refrigerated storage, the greatest mean pH decline rate and acidity increase rate as well as the lowest final pH and acidity were related to 8I. Treatment I was in contrast to 8I for mentioned parameters. An interesting point regarding the sequence of inoculation was adaptation ability of starter bacteria in treatments with higher rate of inoculation before fermentation. Treatment 8I had higher pH drop rate and acidity increase rate than (4+4)I, and the latter than (2+6)I. It is apparent that adding starter bacteria at the end of fermentation (after fermentation instead of before fermentation) to the medium with low pH and high acidity could imply pH and acid shocks to them (4, 15, 16), leading lower activity and slower pH drop and acidity increase during storage period. This fact is well-known as ‘stress adaptation’ phenomenon in microbiology texts. According to Table 4, the greatest amounts of acetic acid at the end of fermentation as well as at the end of refrigerated storage were related to the treatments 8I. In contrast, the lowest amounts were observed for standard inoculation (I) and the 2-fold inoculations before fermentation. 

*Viability of probiotic microorganisms at the end of fermentation and during storage*

Table 5 indicates viable counts of probiotic bacteria in different treatments at the end of fermentation as well as during 21 days of storage time. According to this Table, following descending relation was significantly observed among treatments in viability of both probiotics at the end of fermentation:

8I > (4+4)I > 4I > (2+6)I > (2+2)I > (1+3)I > I 

This relation confirmed the stress adaptation rule (Section 3.1). Inoculation of starter bacteria to the medium with considerably smaller exposure to detrimental factors (*e.g*., pH, acidity, redox potential, hydrogen peroxide, flavor agents and bacteria competitions) would enable the bacteria to retain their survival more efficiently due to better adaptation (2). This is the reason that the treatment (4+4)I possessed higher viability of probiotics than (2+6)I and the treatment 4I than (2+2)I and then, (1+3)I. According to Table 5, bifidobacteria showed significantly greater viability than *L. acidophilus *at the end of fermentation and throughout the storage time in all treatments. This could be attributed to the initial higher population of bifidobacteria in ABY-type culture mix inoculum as well as to the greater resistance of these bacteria compared to *L. acidophilus*. This observation was in conformity with previous researches (6, 9). *L. acidophilus *had significantly greater viability loss compared to other probiotic throughout the storage period (data not shown).

During the first 7 days of refrigerated storage, the treatments contained highest and lowest viabilities were the same as those at the end of fermentation. At d 14, for *L. acidophilus*, the greatest viabilities after the treatment 8I were related to (4+4)I and (2+6)I (statistically the same), and then, 4I and (2+2)I. For bifidobacteria, after 8I, treatments (4+4)I and (2+6)I (statistically the same) were placed. At d 14, the first record from viable counts was dedicated to I and (2+6)I and then, (4+4)I. The same rank for bifidobacteria was first for (2+6)I and then, respectively to (4+4)I and 8I. These observations indicated that the treatments with more amounts of inoculation before fermentation presented greater viability at the end of fermentation and at the early days of refrigerated storage period. However, afterwards, the other treatments (more amounts of inoculation after fermentation) became overcome because the bacteria that withstand detrimental factors in a shocking exposure are strong and resistance enough to possess significantly greater growth and activity during the rest of storage time compared to those gradually adapted during fermentation. Therefore, even though the viable population of probiotic cells added to the product at the end of fermentation immediately decreased considerably, those withstand the harsh conditions were highly tolerable and can growth and being active in media, leading less loss of cells survival and higher viability. The highest and lowest viability losses during the first 7-day of storage were related to treatments with inoculation before fermentation (4I, 8I and I) and (2+6)I, respectively. The latter treatment maintained its record to the end of storage time (data not shown). Another reason for cell loss in treatments with higher initial inoculation level before fermentation (8- or 4-fold) could be significantly lower final pH and higher final titrable acidity at the end of fermentation (Table 4) that make media situations more detrimental to probiotics during storage time.

## Conclusion

This work demonstrated that inoculation level and sequence significantly and considerably affects the viability of *L. *acidophilus and bifidobacteria at the end of fermentation as well as during the refrigerated storage period. Overall, greater incubation level led to higher probiotic viability at the end of fermentation and during the early days of storage period, but to an inverse impact during the rest time of storage. Exposure of probiotic cells to detrimental environmental conditions at the end of fermentation in a shocking state alternatively resulted in significant loss and retain of viability at early stages of storage and at the rest of storage time, respectively. 
